# Tumor necrosis factor antagonists for paradoxical inflammatory reactions in the central nervous system tuberculosis: Case report and review

**DOI:** 10.1097/MD.0000000000022626

**Published:** 2020-10-23

**Authors:** Miguel Santin, Cristina Escrich, Carles Majòs, Mariona Llaberia, Maria D. Grijota, Imma Grau

**Affiliations:** a Tuberculosis Unit, Service of Infectious Diseases, Bellvitge University Hospital-Bellvitge Institute for Biomedical Research (IDIBELL); b Department of Clinical Sciences, University of Barcelona; L’Hospitalet de Llobregat; c Service of Internal Medicine, Hospital Verge de la Cinta; Tortosa; d Department of Neuroradiology, Bellvitge University Hospital-Bellvitge Institute for Biomedical Research (IDIBELL); e Service of Infectious Diseases, Bellvitge University Hospital-Bellvitge Institute for Biomedical Research (IDIBELL), Spain.

**Keywords:** adalimumab, case report, central nervous system tuberculosis, immune reconstitution inflammatory syndrome, infliximab, intracranial tuberculomas, paradoxical reaction, thalidomide, TNF antagonists

## Abstract

**Rationale:**

Paradoxical reaction/immune reconstitution inflammatory syndrome is common in patients with central nervous system tuberculosis. Management relies on high-dose corticosteroids and surgery when feasible.

**Patient concern:**

We describe 2 cases of HIV-negative patients with corticosteroid-refractory paradoxical reactions of central nervous system tuberculosis.

**Diagnoses:**

The 2 patients experienced clinical impairment shortly after starting therapy for TB, and magnetic resonance imaging showed the presence of tuberculomas, leading to the diagnosis of a paradoxical reaction.

**Interventions:**

We added infliximab, an anti-tumor necrosis factor (TNF)-alpha monoclonal antibody, to the dexamethasone.

**Outcomes:**

Both patients had favorable outcomes, 1 achieving full recovery but 1 suffering neurologic sequelae.

**Lessons:**

Clinicians should be aware of the risk of paradoxical reactions/immune reconstitution inflammatory syndrome when treating patients with tuberculosis of the central nervous system and should consider the prompt anti-TNF-α agents in cases not responding to corticosteroids.

## 1 Introduction

Clinical deterioration following initial improvement with anti-tuberculosis therapy in central nervous system tuberculosis (CNS-TB) is a serious complication causing high mortality and disability.^[1]^ This paradoxical reaction (PR) is particularly common in patients infected with the human immunodeficiency virus (HIV); the paradoxical immune reconstitution inflammatory syndrome (IRIS), which is triggered by starting antiretroviral therapy and the secondary immune restoration.^[2,3]^ Treatment of PR/IRIS comprises the administration of high doses of corticosteroids, with or without surgery, but success rates are non-well known.

High levels of tumor necrosis factor (TNF)-α, a cytokine that plays a central role in acute inflammation and granuloma formation,^[4]^ have been found in the cerebrospinal fluid (CSF) of patients with CNS-TB who develop PR/IRIS.^[3,5]^ As such, there has been an increase in the number of case reports of CNS-TB with PR/IRIS that have been successfully treated with anti-TNF antagonists.^[6–22]^ In this article, we report 2 cases of CNS-TB that developed PR and were treated successfully with infliximab and review the literature on the use of TNF-α antagonists for PR/IRIS associated with CNS-TB.

## 2 Case reports

### 2.1 Case 1

A 53-year-old, HIV-negative female from Dominican Republic who had been diagnosed with sarcoidosis (affecting the lungs, lymph nodes, and kidneys) 2 years earlier and receiving immunosuppressive therapy with tacrolimus and mycophenolate, who was admitted to another hospital in July 2015 because of lymphocytic meningitis and a high adenosine deaminase (ADA) level. *Mycobacterium tuberculosis* grew in both a CSF and a bronchoalveolar aspirate (BAS). Magnetic resonance imaging (MRI) of the brain without gadolinium contrast revealed no relevant findings. Treatment was initiated with rifampin, isoniazid, pyrazinamide, and ethambutol, plus dexamethasone 16 mg/day. Six weeks later, susceptibility testing showed resistance to isoniazid, which was substituted for moxifloxacin. Cycloserine was also added and tapering of the dexamethasone dose was started.

**Figure 1:**
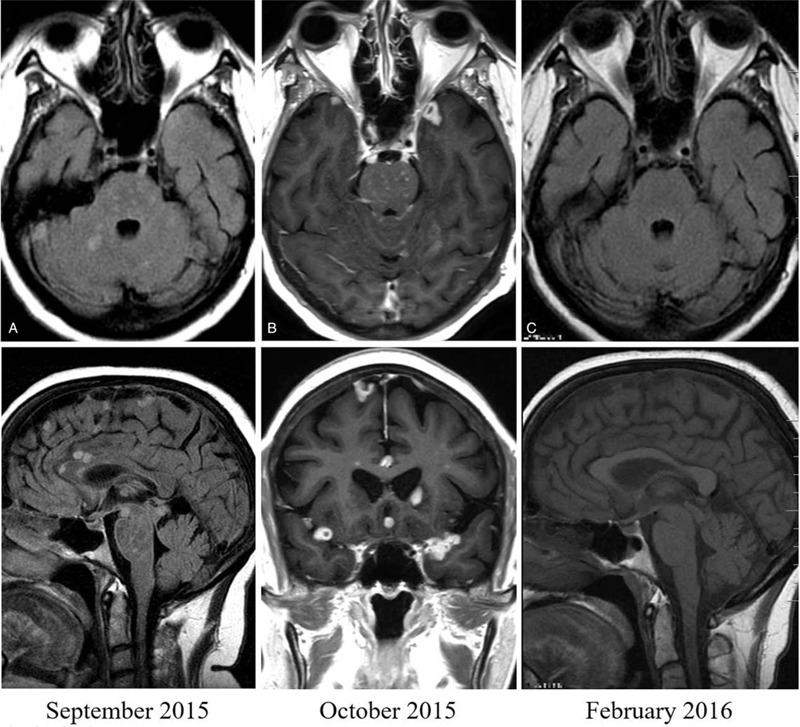
Magnetic resonance imaging of case 1. Axial and sagittal fluid-attenuated inversion recovery images without gadolinium (A) and axial and coronal T1-weighted images with gadolinium (B) performed 5 (September 2015) and 8 weeks (October 2015) respectively after starting tuberculosis treatment, showing multiple supra-and infratentorial lesions, distributed in the brain parenchyma and subarachnoid space. Axial and sagittal scans without gadolinium (C), taken after 6 weeks after receiving a three-dose course of infliximab, showing complete resolution of the tuberculomas.

Five weeks later, in September 2015, she presented with a generalized tonic-clonic seizure. A second MRI without gadolinium contrast showed multiple infra- and supra-tentorial lesions consistent with tuberculomas (Fig. 1A), plus an infarct of the basal ganglia (not shown). CSF analysis showed a shift toward polymorphonuclear cell predominance, and culture for mycobacteria was negative. Cycloserine was stopped and the high doses of dexamethasone were restarted. Following slight improvement in her clinical condition, the patient was referred to our hospital.

On admission, the patient was oriented and alert. She reported having a headache and instability when walking. She had slight dysmetria in the finger-to-nose test, dysdiadochokinesis, distal tremor and was unable to perform tandem gait. An MRI with gadolinium on admission revealed multiple (>50) supra- and infratentorial lesions distributed in the brain parenchyma and subarachnoid space. There was evidence of leptomeningeal enhancement involving cranial nerves III, V, VI, VII, both cerebral middle cerebral arteries, the prepontine and interpeduncular cisterns, the vermis and cerebellar folia, and the mild-enlarged ventricular system (Fig. 1B). CSF analysis showed a white blood cell (WBC) count of 380/mm^3^ (74% lymphocytes); protein, 169 mg/dL; glucose, 30 mg/dl; and ADA 0.35 μkat/L (normal <0.15 μkat/L). A Polymerase chain reaction (PCR) for *M. tuberculosis*, performed with the Xpert MTB/RIF (Cepheid, Sunnyvale, USA), and culture were negative. After an attempt with higher doses of dexamethasone (24 mg/d) and rifampin 900 mg/d plus linezolid 600 mg/day without improvement, infliximab 300 mg/kg was tried, and the patient made a dramatic recovery within the first 48 hours of therapy. Two additional doses of infliximab were given at 2 and 6 weeks apart. The MRI without gadolinium contrast, taken at the original hospital following all 3 doses of infliximab, showed complete resolution of the tuberculomas (Fig. 1C), but with residual infarct in the vermis and basal ganglia. Dexamethasone was subsequently tapered, and she completed a 12-month course of anti-tuberculosis treatment. In September 2017, she suffered a subarachnoid hemorrhage due to rupture of an aneurysm at the bifurcation of the right middle cerebral artery, but there was no evidence of active TB. Two years later she underwent kidney transplant and she is currently under immunosuppressive therapy.

### 2.2 Case 2

A 19-year-old, HIV-negative male from Pakistan who was being evaluated in another hospital because of fever and enlarged abdominal lymph nodes, and who was subsequently admitted in September 2017 because of headaches and fever. The initial CSF exam showed a WBC count of 304/mm^3^ (96% lymphocytes); protein, 289 mg/dl; glucose 46.9 mg/dl; and ADA 0.33 μkat/L. A PCR for *M. tuberculosis* (Xpert MTB/RIF [Cepheid, Sunnyvale, USA]) was negative, as was the culture for mycobacteria. The QuantiFERON-TB Gold In-Tube assay (Qiagen, Hilden, Germany) was positive. With the presumptive diagnosis of disseminated and CNS-TB, treatment with rifampin, isoniazid and ethambutol plus Dexamethasone was initiated. His clinical condition deteriorated over the following 24 hours and he was referred to our hospital.

**Figure 2:**
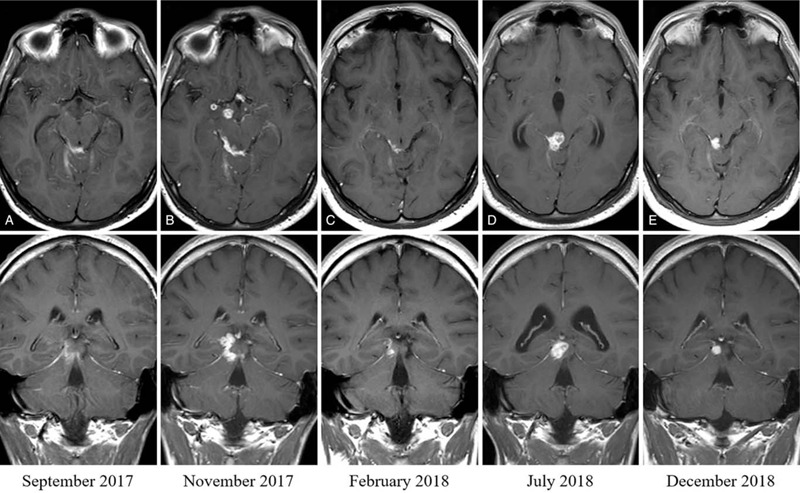
Axial and coronal contrast-enhanced T1-weighted magnetic resonance images of case 2. At diagnosis (September 2017): extraparenchymal enhancing lesion on the right ambient cistern infiltrating the right superior colliculus (A). Six weeks later (November 2017): appearance of new tuberculomas in midbrain tegmentum, suprasellar cisterns and right temporal uncus (B). After 3 weeks of a three-dose course of infliximab (February 2018): Remarkable reduction of tuberculomas and leptomeningeal enhancement (C). On the eleventh month of tuberculosis treatment (July 2018): obstructive hydrocephalus secondary to mass effect over Silvius aqueduct (D). And 3 months after discontinuation of tuberculosis treatment (December 2018): Mild leptomeningeal enhancement, with resolution of the mass effect (E).

On hospital admission, the patient was drowsy but was responsive to verbal stimuli, and he had neck stiffness and diplopia. The MRI showed leptomeningeal enhancement, predominantly at the basal cisterns, and enhancement of the III, V, VI, VII, and VIII cranial nerves (Fig. 2A). Study of the CSF for syphilis, brucella, and cryptococcus were negative, as were as conventional cultures, and cytological examination revealed no atypical cells. A HIV ELISA test was negative. Moxifloxacin was added to his treatment according to the protocol for initial treatment of CNS-TB at our institution, and he was discharged under treatment for TB plus dexamethasone 12 mg/day.

Four weeks later, the patient presented with unsteady gait and weakness of his right lower limb. The MRI showed global impairment of the initial leptomeningeal enhancement, new parasagittal frontal brainstem tuberculomas (Fig. 2B), and left pontine and right thalamic infarcts. CSF exam showed no relevant changes, except in his WBC (50% polymorphonuclear). Given the neurologic impairment despite adjunctive dexamethasone, we gave a 3 mg/kg dose of infliximab, which resulted in clinical improvement of his gait. He was discharged pending the administration of 2 more doses of infliximab. An MRI 6 weeks after the last dose of infliximab showed a marked reduction in both the leptomeningeal enhancement and the tuberculomas (Fig. 2C).

**Figure 3:**
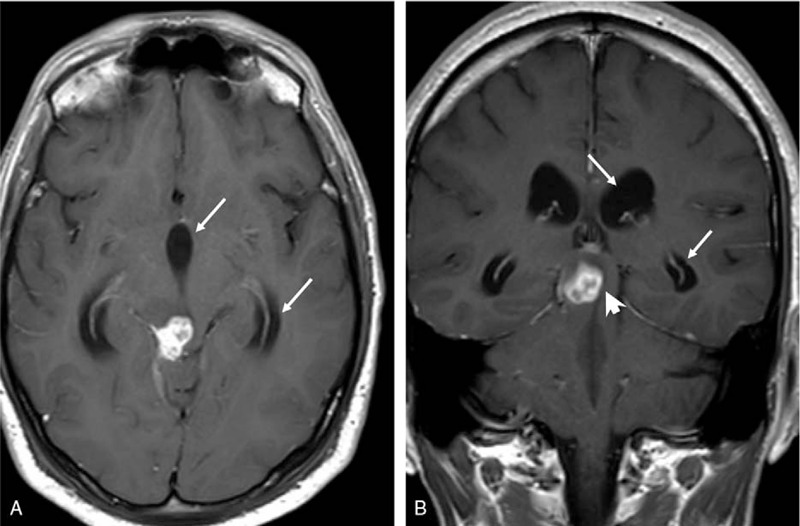
Detailed axial (A) and coronal (B) contrast-enhanced T1-weighted magnetic resonance images showing enlargement of III ventricle and temporal lateral ventricle horns (arrows) due to obstructive hydrocephalus secondary to mass effect over Silvius aqueduct (arrowhead).

A few weeks later, when tapering the dexamethasone dose, he developed dizziness and right ear deafness. Otorhinolaryngology consultation revealed that he had suffered an irreversible lesion of the right VIII cranial nerve. In July 2018, on the eleventh month of TB treatment, and 1 month after dexamethasone had been discontinued, a follow-up MRI showed obstructive triventricular hydrocephalous secondary to a tuberculoma blocking the aqueduct of Silvius (Fig. 2D, Fig. 3A, and 3B). A second course of 3 doses of infliximab was administered, and the MRIs performed after treatment showed complete resolution of the hydrocephalous and the near-disappearance of the tuberculoma (Fig. 2E). He completed a 12-month course of treatment for the TB, and in March 2020 (18 months after stopping treatment), the patient is cured with residual deafness of the right ear.

## 3 Literature review

We conducted a systematic review looking for cases of PR/IRIS associated with CNS-TB that had been treated with TNF-α antagonist. We searched the NCBI databases using the following search strategy: “central nervous system tuberculosis OR intracranial tuberculoma OR meningeal tuberculosis AND immune reconstitution inflammatory syndrome OR immune reconstitution syndrome OR paradoxical reaction AND infliximab OR adalimumab OR etanercept OR thalidomide.” We also reviewed citations of the main articles to retrieve additional publications, and we excluded citations for non-human studies, reviews, and opinion articles.

We identified 17 publications reporting 42 cases; of these, 26 were reported in case reports or case series^[6–19,21,22]^ and 16 were reported in a single prospective observational study.^[20]^ We excluded one case that was reported in 2 publications, resulting in 41 cases being included (Table 1). Here we describe the demographics, PR/IRIS characteristics, treatment, and main outcomes of the 43 cases (41 from the literature and the 2 reported here) (Table 2). In total, 13 were adults treated with the monoclonal anti-TNF-α antibody antagonists infliximab, adalimumab, or thalidomide, and 30 were children treated with thalidomide. All but 2 patients (1 adult and 1 child) were treated with corticosteroids as adjunctive treatment to anti-TB therapy. The regimens used for PR/IRIS treatment varied greatly in dose and duration. Infliximab was given at doses ranging from 2.5 to 10 mg/kg, 3 doses one month apart (4 cases), 1 initial dose followed by 2 additional doses 2 and 4 weeks apart (2 cases), a single dose (1 case) and the dose and duration were unknown in one case. Adalimumab was given at a dose of 40 mg 2 weeks apart, 2 (1 case) and 4 weeks apart (1 case). Regarding thalidomide, doses ranged from 1 to 24 mg/kg/day (27 cases), and 100 mg given twice a day (1 case) or once a day (1 case). The duration of thalidomide treatment was consistently higher than that of either infliximab or adalimumab, ranging from 2 to 9 months (median 5 months) in 28 cases (one patient died after 2 weeks of treatment).

**Table 1:**
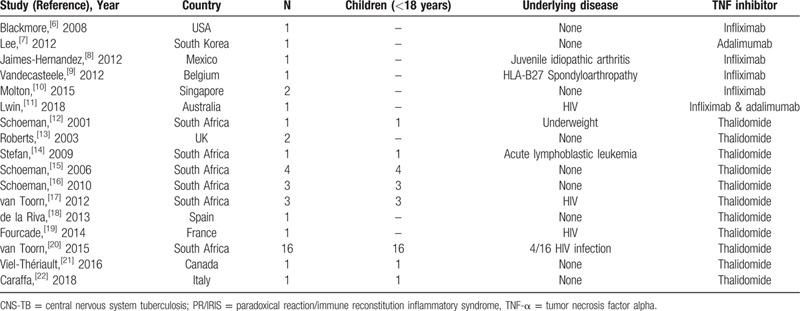
Summary of the 17 publications on paradoxical reaction/immune reconstitution inflammatory syndrome of central nervous system tuberculosis treated with TNF-α antagonists.

**Table 2:**
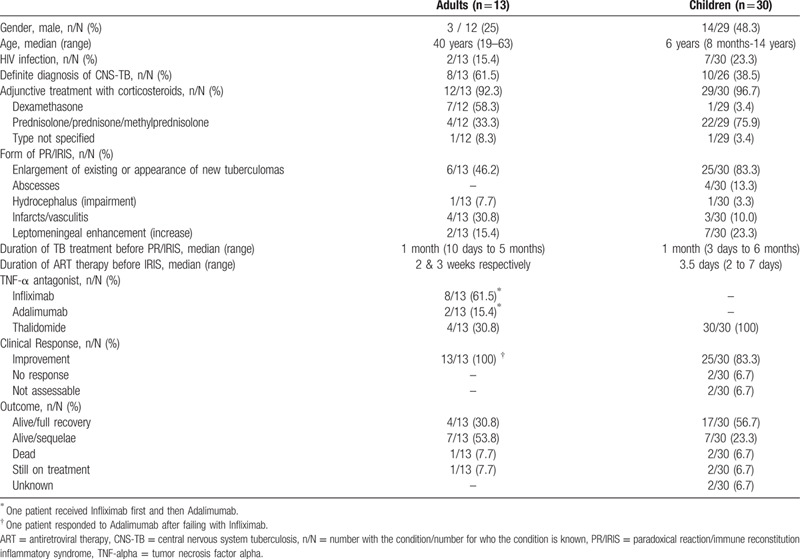
Characteristics and outcomes of paradoxical reaction/immune reconstitution inflammatory syndrome associated with central nervous tuberculosis in 43 patients treated with TNF-α antagonists.

Overall, 38 (83.5%) cases improved, of which 13 were adults and 25 were children. Although most children improved progressively within weeks to months after starting therapy, 2 children did not respond and we could not ascertain the clinical response in another 2. Twenty four children (80.0%) survived and 56.7% experienced full recovery. Among the 13 adults, 11 (84.6%) were alive after treatment or a variable follow-up, but 53.8% of these were cured with sequelae of some type. One patient who initially responded died after 9 months from an intracerebral hemorrhage secondary to rupture of friable arterial collaterals.

## 4 Discussion

This review shows that there is encouraging evidence on the potential benefit of TNF-α antagonists for managing PR/IRIS in CNS-TB, without compromising microbiological cure. Indeed, treatment with these agents improved the clinical conditions of most patients after unsuccessful treatment with corticosteroids.

Much of the mortality and disability caused by CNS-TB can be attributed to the harmful effect of the immune-mediated inflammatory response of the host trying to contain *M. tuberculosis*. Inflammation is believed to be enhanced by the exposure of excess *M. tuberculosis* antigens to the immune system during anti-TB therapy, which in turn would cause a disproportionate response and its clinical expression in form of PR.^[23,24]^ In HIV co-infected patients, starting antiretroviral therapy will trigger this response in a patient receiving TB treatment. It is entirely plausible that amplification of the inflammatory response takes place in most patients, if not all, and that PR/IRIS represents an extreme of this clinical expression. Whereas corticosteroids would be sufficient to control the inflammatory response in most patients, rendering the process clinically silent, others would develop a more extreme clinical syndrome of PR/IRIS in which corticosteroids alone are insufficient. In fact, although adjunctive treatment with corticosteroids has been shown to reduce mortality in patients with CNS-TB,^[25]^ PR/IRIS still occurs in 31.2% to 56.0% of non-HIV-infected^[26,27]^ and in 47% HIV-infected patients.^[28]^

The pathogenicity of the PR/IRIS is poorly understood. TNF-α, a pro-inflammatory cytokine, is mainly produced by monocytes and macrophages and has a central role in the immune response to *M. tuberculosis* by recruiting immune cells, enhancing the anti-TB activity of macrophages, and promoting granuloma formation.^[4]^ It has been postulated that sustained immune activation in the CSF causes the disproportionate inflammatory response that leads to PR/IRIS. Consistent with this, higher levels of TNF-α have been found in the CSF of patients with CNS-TB compared with that of healthy controls,^[5]^ and research has shown that the TNF-α to interferon gamma ratio in CSF is highly predictive of IRIS in HIV patients with CNS-TB.^[28]^ Furthermore, thalidomide, a potent TNF-α produced by stimulated monocytes, has been shown to reduce TNF-α levels in the CSF of rabbits with TB meningitis.^[29]^ Therefore, the putative mechanism of action of anti-TNF agents in PR/IRIS would seem straightforward, and reduction of TNF-α levels in the CSF may be expected. However, available data give conflicting results. In a prospective study of CNS-TB in HIV-negative patients, there was no correlation between either basal TNF-α levels or changes in CSF TNF-α levels and the development of PR.^[30]^ In addition, in a clinical trial of CNS-TB in children, adjunctive thalidomide did not significantly lower TNF-α levels in the CSF compared to placebo.^[31]^ Because thalidomide is also a co-stimulator of T-cells, which also produce TNF-α, its immunomodulatory effect may be driven by achieving a balance between inhibiting monocyte and macrophage cytokines and its co-stimulating effect on the T cells.^[32]^ Unfortunately, there are no data on CSF cytokine levels in patients with PR/IRIS associated with CNS-TB treated with anti-TNF-α monoclonal antibodies.

What the role of TNF-α antagonists in the management of CNS-TB should be is currently unknown. However, given the major difficulties posed by PR/IRIS despite high doses of corticosteroids, anti-TNF-α monoclonal antibodies would appear to offer a reliable alternative. It is possible that their safety profiles may even overcome the secondary effects associated with repeated high doses of corticosteroids.

To date, the strategy of using adjunctive TNF-α antagonists instead of corticosteroids to prevent CNS-TB-associated PR/IRIS and improve outcomes has only been explored with thalidomide in children with stage 2 and 3 TB meningitis. In an open-label study with escalating thalidomide doses (6, 12, and 24 mg/kg/day) in South Africa,^[33]^ clinical and neuroimaging outcomes were better than previously observed with historical controls, and the thalidomide was well tolerated. The same group later conducted a randomized, double-blinded, placebo-controlled trial using doses of 24 mg/kg/day.^[32]^ However, the study was terminated prematurely because all adverse events and deaths occurred in the thalidomide arm. The authors concluded that the results did not support the use of high doses of thalidomide as adjunctive therapy for TB meningitis in children. There have been no similar studies among adults with CNS-TB, either using thalidomide or using anti-TNF-α monoclonal antibodies, and this is an area that warrants further research.

In conclusion, there is encouraging evidence on the potential value of TNF-α antagonists in the management of CNS-TB and deserves further evaluation in clinical trials.

## Author contributions

**Cases review and summary:** Miguel Santin, Cristina Escrich, Immaculada Grau, Maria D. Grijota, and Mariona Llaberia.

**Conceptualization:** Miguel Santin.

**Magnetic resonance imaging review:** Carles Majòs.

**Methodology:** Miguel Santin.

**Systematic review:** Miguel Santin.

**Writing – original draft:** Miguel Santin and Carles Majòs.

**Writing – review & editing:** Miguel Santin, Cristina Escrich, Carles Majòs, Immaculada Grau, Maria D. Grijota, and Mariona Llaberia.
